# Experiences with risk-reducing mastectomy in Norwegian *BRCA1/2* carriers without prior breast cancer

**DOI:** 10.1007/s10689-025-00484-6

**Published:** 2025-07-29

**Authors:** Hanne Kjensli Hyldebrandt, Astrid Tenden Stormorken, Valeria Vitelli, Amy Østertun Geirdal, Eli Marie Grindedal

**Affiliations:** 1 Department of Medical Genetics, Oslo University Hospital, Oslo, Norway; 2 Institute of Clinical Medicine, University of Oslo, Oslo, Norway; 3 Department of Biostatistics, Oslo Centre for Biostatistics and Epidemiology, Institute of Basic Medical Sciences, University of Oslo, Oslo, Norway; 4 Department of Social Work, Child Welfare, and Social Policy, Faculty of Social Sciences, OsloMet– Oslo Metropolitan University, Oslo, Norway

**Keywords:** *BRCA1*, *BRCA2*, Risk-reducing mastectomy, Decision-making, Satisfaction

## Abstract

**Background:**

*BRCA1* and *BRCA2* carriers without prior breast cancer have the option to undergo risk-reducing mastectomy (RRM). The aim of this study was to investigate factors associated with their decision, experiences with the process, and satisfaction with the procedure.

**Materials and methods:**

We distributed a cross-sectional survey to 425 women aged 25–70 with a pathogenic *BRCA1/2* variant. The survey collected data on sociodemographic factors, family cancer history, reasons for choosing RRM or not, experiences with the decision-making process, support from healthcare, and surgery satisfaction. Multivariate logistic regression analysis identified factors associated with the decision to undergo RRM and satisfaction with having undergone surgery.

**Results:**

Of the 272 respondents, 190 (69.9%) had undergone RRM. Having children and being a *BRCA1* carrier were both associated with higher probability of choosing RRM, with an OR of 3.5 (*p* = 0.005 and *p* < 0.001 respectively). Among those who had undergone RRM, 78.9% (150/190) were satisfied with their decision and would choose the same procedure again. Feeling satisfied with support from the health care system gave an OR of 5.5 for being satisfied with having undergone RRM (*p* < 0.01). Those who found the decision difficult had lower odds of being satisfied (OR 0.2, *p* = 0.02).

**Conclusion:**

Being a *BRCA1* carrier and having children were strongly associated with choosing RRM. Most participants felt relieved after RRM, were satisfied with their decision, and would choose the surgery again. Support from healthcare providers during decision-making was linked to higher satisfaction with having undergone surgery while those who struggled with the decision reported lower satisfaction.

**Supplementary Information:**

The online version contains supplementary material available at 10.1007/s10689-025-00484-6.

## Background

Women with germline pathogenic variants in *BRCA1* or *BRCA2* (*BRCA1/2* carriers) have about a 70% lifetime risk of developing breast cancer (BC) [1]. In Norway, they are offered annual mammography and MRI from the age of 25. From the same age, they may also choose risk-reducing mastectomy (RRM), which will reduce their risk of developing BC by 90–95% [2]. With increasing access to genetic testing (GT), more female *BRCA1/2* carriers are identified before being diagnosed with BC and ovarian cancer (OC), and they must decide whether and when to undergo surgery.

A previous study from Norway has demonstrated that 74% of *BRCA1/2* carriers who had undergone RRM, felt relieved after the procedure and 86% would have chosen the same procedure again [3]. A later study from Norway found that *BRCA1/2* carriers had a health-related quality of life (QoL) comparable to age-matched female population [4]. In these two studies, 49% and 21% of the participants had BC. Contradictory findings are reported regarding how the indication for surgery (risk reducing, therapeutic or both) may have an impact on levels of satisfaction. A British study found that women who underwent RRM without a BC diagnosis had a lower median satisfaction following surgery compared to those with a diagnosis of BC [5]. Contrary to these findings, a recent German study of long-term satisfaction and health-related QoL in *BRCA1/2* carriers who had undergone RRM and reconstruction, found that women without a prior BC diagnosis had higher mean satisfaction scores in terms of both psychosocial, sexual, and physical well-being [6].

In a recent study on uptake of RRM in Norwegian *BRCA1/2* carriers without prior BC, we found that 55% of *BRCA1* and 45% of *BRCA2* carriers had chosen surgery. *BRCA1* carriers had surgery at a younger age and at a shorter time interval following GT. Of those tested between 25 and 29 years, 40% of *BRCA1* carriers and only 8% of *BRCA2* carriers had undergone RRM within 5 years after GT [7].

While the uptake of surgery in healthy carriers is known to vary between *BRCA1* and *BRCA2* carriers and across age groups [7], limited knowledge exists regarding other factors that are associated with the decision of when and whether to undergo RRM. There is also limited knowledge on how they have experienced the decisional process, the help they have received from the health care system and whether these factors have affected their satisfaction with surgery. The aim of this study was therefore to investigate these issues in a cohort comprised only of *BRCA1/2* carriers without prior BC. As many previous studies of satisfaction with surgery have included women with and without BC, we also wanted to investigate overall satisfaction with RRM in this cohort of healthy *BRCA1/2* carriers.

## Material and methods

Section for Hereditary Cancer at Oslo University Hospital (OUH) has provided genetic counseling and testing for hereditary cancer since the mid-1990s. All patients diagnosed with a pathogenic variant are offered inclusion in the section’s consent-based registry. For this study, female *BRCA1/2* carriers aged 25–70 were identified from the registry up to April 17, 2023. Eligibility criteria included no prior diagnosis of BC or OC and no active treatment for other cancers. Four hundred and twenty-five *BRCA1/2* carriers, 263 *BRCA1* and 162 *BRCA2,* fulfilled the inclusion criteria.

A cross-sectional study based on a study specific questionnaire was designed. The eligible participants were sent an electronic questionnaire that included questions on whether they had chosen to undergo RRM, factors that had influenced their decision-making process (supplementary Tables 1 and 2), and their experiences with the information and support they received from the healthcare system during this process (Supplementary Table 3). The women were asked to rate their overall satisfaction with the help/information/support they received from health care professionals during the decision-making process on a scale from 1 to 10. We also asked whether their breasts were important for their self-esteem and if they were satisfied with their breasts. The authors for this study specifically developed these questions.

In the second part of the questionnaire, participants who had chosen RRM, answered the same questionnaire as used in our study from 2014 [3], regarding their satisfaction with having undergone surgery and their experiences with the procedure (Supplementary Table 4). However, for those who answered that they would not have chosen the same operation again, the following changes were made in the question regarding what they would choose instead: in addition to the alternatives “screening” and “RRM but without reconstruction”, we added the alternatives “other type of implant” and “other”. We also asked them to rate their overall satisfaction with having undergone surgery on a scale from 1 to 10. Subsequently, the results were dichotomized so that those who answered 1–5 were categorized as "not satisfied" and those who answered 8–10 were categorized as "satisfied”, while those who answered 6–7 were categorized as “neither/nor”.

Sociodemographic data, both current and at the time of RRM were collected: Age (continuous), employment, having children, marital status (married/in a relationship, or single), and education (≤ 13 years or > 13 years of education). Data on family history of cancer was also collected: Having a mother or sister with BC or OC, and a mother or sister dead of BC or OC.

### Ethics

The study was approved by the Regional Ethics Committee (REC) (Reference number 395119).

### Statistics

Frequencies (%) and means were used for presenting the descriptive data. *T*-test and Pearson’s chi-square test was used for the statistical comparison of the two groups (performed RRM and not performed RRM). A *p*-value of less than 0.05 was regarded as significant. Odds ratios (ORs) and 95% confidence intervals (CIs) for choosing RRM were estimated using multivariate logistic regression analysis with the following independent variables: mutation status (*BRCA1 or BRCA2*), age, having children (yes/no), education, employment status, having a sister and/or mother with BC or OC and marital status. For women who had not undergone RRM, we used their status at the time of inclusion for the variables age, having children, employment status and marital status. For those who had undergone RRM, we used their status at the time of RRM for the same variables. Marital status was dichotomized to “in a relationship” and “living alone”. Education was dichotomized to “up to 13 years of education” and “more than 13 years of education”.

To examine whether the women’s satisfaction with having undergone RRM was related to their experience of the decision-making process, multivariate logistic regression analysis was conducted. The considered outcome was “Satisfied with having undergone RMM”, where a positive outcome was defined as having answered “8, 9 or 10”, while a negative outcome of “Not satisfied” was defined by having answered “1–5”. Women who answered “6 or 7” were thus not included in this multivariate logistic regression analysis, to ensure a robust assignment to either the positive or negative outcome groups. Independent variables were “satisfied with information and help from the healthcare during the decision-making process”, “satisfied with information and help from geneticists during the process”, “satisfied with information and help from surgeon during the process”, and “if the decision was difficult”, all binary categorical variables with levels yes/no.

To assess the impact of the chosen cut points and the exclusion of respondents scoring 6 or 7, sensitivity analyses were conducted using several alternative cutoffs. Logistic regression analyses were performed when defining the outcome in the following alternative ways: (1) scores of 7–10 as "satisfied" and 1–6 as "not satisfied," and (2) scores of 6–10 as "satisfied" and 1–5 as "not satisfied" (Supplementary Table 5).

The statistical analyses were conducted using STATA version 18.

## Results

A total of 272 (272/425 = 64.0%) answered the questionnaire, 168/263 (63.9%) *BRCA1* carriers and 104/162 (64.2%) *BRCA2* carriers. Mean age at inclusion was 48.5 years. Sociodemographic data for the total cohort and the two genes separately are summarized in Table 1.

**Table 1:** Sociodemographic data in total cohort and by gene

	Total cohort (n = 272)	*BRCA1* (n = 168)	*BRCA2* (n = 104)	*p*-value
Mean age at inclusion (range)	48.5 (25–70)	49.0 (25–70)	47.8 (26–70)	*p* = 0.42
Mean age at genetic testing (range)	37.7 (16–70)	36.5 (18–70)	39.6 (16–68)	*p* = 0.03
Number of women who have undergone RRM	190 (69.9%)	133 (79.2%)	57 (54.8%)	*p* < 0.001
Mean age at RRM (range)	42.6 (24–67)	41.6 (24–65)	43.5 (26–67)	*p* = 0.41
*Marital status*				
Married/in a relationship	224 (82.4%)	135 (80.4%)	89 (85.6%)	*p* = 0.27
Single	48 (17.6%)	33 (19.6%)	15 (14.4%)	
Employment	212 (78.0%)	133 (79.2%)	79 (76.0%)	*p* = 0.54
*Education*				
≤ 13 yrs	86 (31.7%)*	56 (33.3%)	30 (29.1%)**	*p* = 0.47
> 13 yrs	185 (68.3%)*	112 (66.7%)	73 (70.9%)**	
Having children	220 (80.9%)	135 (80.4%)	85 (81.7%)	*p* = 0.78
Mother/sister with BC/OC	158 (59.0%)***	101 (61.2%)****	57 (55.3%)**	*p* = 0.34
Mother/sister dead due to BC/OC	83 (52.5%)	58 (57.4%)	25 (43.9%)	*p* = 0.10

*RRM* Risk-reducing mastectomy. *BC* Breast cancer. *OC* Ovarian cancer

*n = 271 (One *BRCA2* carrier did not respond). **n = 103. ***n = 268. ****n = 165

Of the total number of respondents, 190 (190/272 = 69.9%) had undergone RRM, while 24 (24/272 = 8.8%) were waiting for the surgery. More *BRCA1* carriers than *BRCA2* carriers had undergone RRM (79.2% vs 54.8%) (*p* < 0.001). *BRCA1* carriers were also younger at GT (*p* = 0.03) (Table 1).

Only 58 of the women (58/272 = 21.3%) had chosen not to undergo RRM. Among these, 19 (19/58 = 32.8%) women, with a mean age of 32.5 years, indicated plans to undergo RRM in the future. Thirty-nine women (39/272 = 14.3%) had no plans of undergoing RRM, the mean age in this group was 55.8 years (range 29–70).

### Sociodemographic factors in women with and without RRM

Among the women who had undergone RRM, a higher proportion were employed (*p* < 0.001), were married (*p* = 0.006), had children (*p* < 0.001), and had a mother or sister who had been diagnosed with BC or OC (*p* = 0.04), compared to those who chose not to undergo RRM (Table 2).

**Table 2:** Sociodemographic data in women with and without risk-reducing mastectomy (RRM)

	Women who have undergone RRM (n = 190)	Women who have chosen not to undergo RRM (n = 58)	*p*-value
Mean age (range)	42.6 (24–67)*	48.0 (25–70)**	*p* = 0.23
*Education*			
≤ 13 years	59 (31.1%)	18 (31.0%)	*p* = 0.98
> 13 years	130 (68.8%)	40 (69.0%)	
Employment	175 (92.1%)	41 (70.7%)	*p* < 0.001
*Marital status*			
Married/in a relationship	171 (90%)	44 (75.9%)	*p* = 0.006
Single	19 (10%)	14 (24.1%)	
Having children	159 (83.7%)	40 (69.0%)	*p* < 0.001
Mother or sister with breast cancer or ovarian cancer	131 (61.2%)***	27 (46.6%)	*p* = 0.04
Mother or sister died from BC/OC	71 (33.2%)***	12 (20.7%)	*p* = 0.07

*Age at RRM, **Age at inclusion, ***n = 214 (190 have undergone RRM + 24 waiting for surgery)

Multivariate logistic regression analysis identified several factors that influenced the likelihood of undergoing RRM. Having children was associated with a higher probability of choosing RRM, with an OR of 3.5 (*p* = 0.005). Furthermore, *BRCA1* carriers had 3.5 times higher odds of choosing RRM compared to *BRCA2* carriers (*p* < 0.001). Having a mother or sister with BC or OC was also associated with a higher likelihood of undergoing RRM, although this result was not statistically significant (OR 1.9, *p* = 0.07). Lastly, the analysis also showed that the likelihood of opting for RRM decreased with increasing age (OR 0.96, *p* = 0.007).

### Self-reported reasons for choosing and not choosing RRM

Compared to the women who opted for RRM, a higher proportion of those who chose not to undergo RRM reported that their breasts were important for their self-esteem (71.5% vs 85.7%, respectively, *p* = 0.05). Additionally, more women in the non-RRM group expressed satisfaction with their breasts, compared to the level of satisfaction reported by women in the RRM group before their surgery (84.5% vs 77.9%, *p* < 0.16).

When asked about their reasons for choosing to undergo RRM, the factor most frequently mentioned by the women was "high likelihood of developing breast cancer" (Table 1). More than 50% also answered, “being recommended the surgery by a geneticist or breast surgeon”, as well as “having a family history of cancer” (Table 3). Many also expressed in the free-text field of the questionnaire, that they did not want to expose their children to the possibility of having a mother with cancer or the fear of dying and leaving their children behind.

**Table 3:** Reasons for choosing risk-reducing mastectomy (RRM)

Statements	Total (n = 214)*	*BRCA1* (n = 147)	*BRCA2* (n = 67)
High likelihood of developing breast cancer	195 (91.1%)	133 (90.5%)	62 (92.5%)
Did not want to undergo breast cancer treatment	90 (42.1%)	62 (42.2%)	28 (41.8%)
Burdensome with breast exams	42 (19.6%)	24 (16.3%)	18 (26.9%)
Fear of dying from breast cancer	97 (45.3%)	64 (43.5%)	33 (49.3%)
Experienced relatives developing breast cancer	111 (51.9%)	75 (51.0%)	36 (53.7%)
Was recommended RRM by a geneticist/breast surgeon	125 (58.4%)	87 (59.2%)	38 (56.7%)
Other reasons	9 (4.2%)	5 (3.4%)	4 (6.0%)

*n = 214 (190 have undergone RRM + 24 waiting for surgery)

Among the women who chose not to undergo RRM, 55.2% answered that they felt safe by attending regular screening (Table 4). Of the 19 women who did not chose to undergo RRM, but indicated plans of undergoing RRM in the future, 11/19 (57.9%) answered that they wanted to undergo RRM when they were done with breastfeeding. The remaining 8 women 8/19 (42.1%) answered that they want to do the surgery when “they reach an age where the cancer risk is high enough”.

**Table 4:** Women who have chosen not to undergo risk-reducing mastectomy (RRM): Reasons for not choosing RRM

Statements	Total (n = 58)	*BRCA1* (n = 21)	*BRCA2* (n = 37)
Feel safe by attending screening	32 (55.2%)	11 (52.4%)	21 (56.8%)
Fear of surgery	10 (17.2%)	4 (19.0%)	6 (16.2%)
Want to breastfeed before potential surgery	15 (25.9%)	4 (19.0%)	11 (29.7%)
Medical reasons	2 (3.4%)	1 (4.8%)	1 (2.7%)

### The decision-making process

**Fig. 1:**
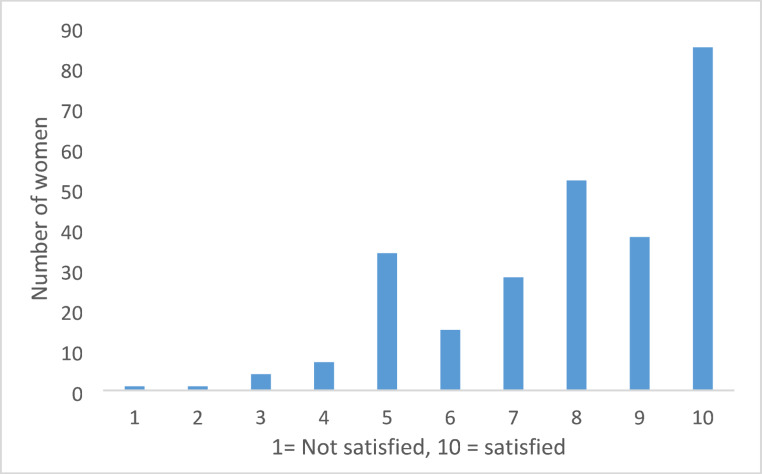
Satisfaction with support from the health care system during the decision-making process

A total of 116 women (116/272 = 42.6%) found the decision to undergo RRM difficult. When asked what was difficult, 62.1% (72/116) answered "deciding *whether* to undergo RRM," while 36.2% (42/116) answered "deciding *when* to undergo RRM”. In terms of decision-making, 89 women (89/272 = 32.7%) reported that they made the decision in consultation with healthcare professionals, while 171 women (171/272 = 62.9%) said they made the decision on their own. Regarding support from geneticists or genetic counselors, 179 (179/272 = 65.8%) women reported receiving adequate information and support, while 28 (28/272 = 10.3%) indicated they did not. The remaining women either responded "don't know" or stated that they did not require any support or information. When asked about support from breast surgeon or plastic surgeon, 159 (159/266 = 59.8%) reported receiving sufficient information and support, while 36 (36/266 = 13.5%) indicated that they did not. Finally, in terms of overall satisfaction with the support received from the healthcare system during the decision-making process, 175 women (175/272 = 64.3%) reported being satisfied (satisfied defined as a score of 8, 9, or 10, as shown in Fig. 1).

### Satisfaction with surgery

**Fig. 2:**
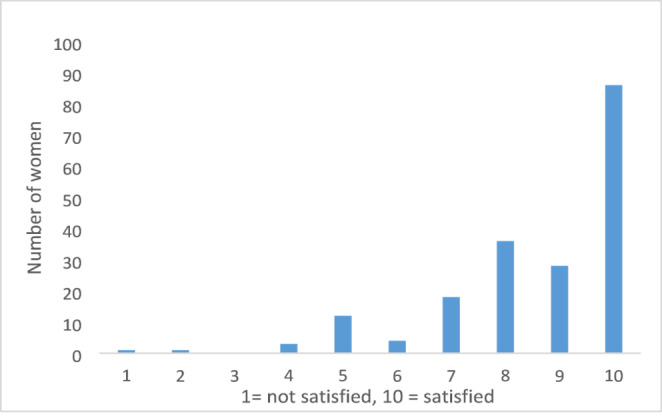
Satisfaction with undergoing risk-reducing mastectomy (RRM)

Of the women who underwent RRM, 163 (163/190 = 85.8%) felt relief after surgery, and 78.9% (150/190) would have chosen the same procedure again. However, only 45% (87/190) stated that the result of the surgery was as expected. Of the 214 women who either had undergone RRM or were waiting for surgery, 18 (8.4%) reported that they did not receive sufficient information before the surgery. Of these, 11 women (11/18 = 61.1%) reported a lack of sufficient information regarding rehabilitation following surgery and 10 (10/18 = 55.6%) indicated that they were not adequately informed about the expected outcomes (Table 5). Despite this, 150 out of the 190 women (78.9%) who had undergone surgery, reported being satisfied with their decision (satisfaction defined as score of 8, 9, or 10, as shown in Fig. 2).

**Table 5:** Risk-reducing mastectomy (RRM) and satisfaction

Sufficient information before RRM (n = 214)*	
Yes	177 (82.7%)
No	18 (8.4%)
Do not know	10 (4.7%)
Waiting for consultation before surgery	9 (4.2%)
If no, would like more information regarding (n = 18)**	
The procedure	6 (33.3%)
Rehabilitation after surgery	11 (61.1%)
The result	10 (55.6%)
Complications	9 (50%)
Other	2 (11.1%)
*Chosen the same operation (n = 190)*	
Yes	150 (79.0%)
No	13 (6.8%)
Do not know	27 (14.2%)
*If no, would have chosen (n = 13)*	
Screening	2 (15.4%)
RRM, but without reconstruction	2 (15.4%)
Other type of implant	6 (46.2%)
Other	3 (23.1%)
*Operative result as expected (n = 190)*	
Yes	87 (45.8%)
No	63 (33.2%)
Do not know	40 (21.1%)
*Relief after RRM (n = 190)*	
Yes	163 (85.8%)
No	8 (4.2%)
Do not know	19 (10%)
Satisfactory size (n = 165)***	
Yes	129 (78.2%)
No	27 (16.3%)
Do not know	9 (5.5%)
Satisfactory shape (n = 165)***	
Yes	93 (56.3%)
No	54 (32.7%)
Do not know	18 (11.0%)
Satisfactory symmetry (n = 165)***	
Yes	107 (64.8%)
No	47 (28.5%)
Do not know	11 (6.7%)

*n = 214 (190 have undergone RRM + 24 waiting for surgery)

**Multiple answers is possible

***Of the 190 women that had undergone RRM, 165 had immediate reconstruction following RRM

Multivariate logistic regression analyses showed that those who were generally satisfied with the support they received from the healthcare system had an OR of 5.5 for being satisfied with having undergone RRM (*p* < 0.01). In contrast, those who found the decision difficult had lower odds of being satisfied (OR 0.2, *p* = 0.02).

## Discussion

Whether and when to undergo RRM is an important decision for *BRCA1/2* carriers both with and without prior BC. In this cohort of carriers without previous BC, we found that only 14% had no plans of undergoing RRM. Having children and being a *BRCA1* carrier were both significantly associated with higher likelihood of choosing RRM. The great majority of those who had undergone surgery felt relieved, were satisfied with their decision and would have chosen the same again. Less than half had found the decision of whether to undergo surgery difficult, and those who did had lower odds of being satisfied. Feeling supported by healthcare providers in the decision-making process was strongly associated with greater satisfaction with having undergone surgery.

Previous studies have also reported that having children and being a *BRCA1* carrier are associated with higher likelihood of choosing RRM [8–11]. The association between having children and choosing RRM is likely because many postpone RRM until after they have completed childbirths and breast-feeding. This is supported by the observation that 58% of those who had plans of undergoing RRM in the future wanted to wait until they were done with breast-feeding. However, in the free-text responses, many of the women who had chosen RRM expressed fear of dying and leaving their children behind as a separate reason for choosing the procedure. Having children may therefore by itself increase the likelihood of choosing RRM.

Our findings of the higher OR of choosing RRM for *BRCA1* carriers compared to *BRCA2* carriers is in line with the results from our study of uptake of RRM from 2024 [7]. In this study, we also observed that *BRCA1* carriers were operated at a younger age and a shorter time after genetic testing [7]. We do not know why more *BRCA1* carriers choose RRM. In the current study, we observed no significant differences between the two groups of carriers regarding marital status, employment, education, parenthood or age at RRM. We observed that a higher proportion of *BRCA1* carriers compared to *BRCA2* carriers had a mother or sister diagnosed with BC or OC, but this difference was not statistically significant. Furthermore, regression analysis did not confirm family history of these cancers as a significant predictor for RRM uptake. Other studies have reported conflicting results regarding the role of family history of BC in the decision to undergo RRM; some found a significant association between family history and RRM [9, 10, 12, 13] while others did not [11, 14]. Several studies have reported that pathogenic variants in *BRCA1* is associated with a high risk of earlier onset and more aggressive BC than *BRCA2* [1]. We therefore cannot exclude that the women are given different information and advice regarding RRM and that this may have had an influence of their choice of undergoing RRM.

In the current and previous studies [3, 15] on patient satisfaction with RRM, the great majority of women were relieved after surgery and satisfied with their decision. However, in the current study including only *BRCA1/2* carriers with no previous BC, we found somewhat higher rates of relief compared to Hagen et al. [3] findings (86% compared to 74%). We also found lower rates of satisfaction with having chosen surgery, compared to both the Hagen- and the Isern study (79% in current study compared to 91% and 100% in previous studies) [3, 15]. The two previous studies included carriers with and without current/previous BC, and lower levels of satisfaction with the decision of surgery in healthy carriers compared to women with BC has also been reported by others [5]. *BRCA1/2* carriers who have or have had BC may feel more motivated for surgery compared to healthy carriers. Moreover, among the women in our cohort who would not have chosen the same operation, only 15% would have chosen not to have surgery and instead follow screening, and 46% would have chosen to be operated but to have a different type of implant. The women with previous or current BC may have had less opportunity to choose different types of implants (silicon or autologous tissue for example) compared to the participants in our study. Moreover, the women included in our study may have had RRM several years ago. If they have experienced complications with implants, this may have arisen after some time and have led them to consider whether they should have chosen a different type of operation.

One of the aims of the current study was to investigate *BRCA1/2* carriers’ experiences with the decision-making process regarding RRM, and whether this is associated with their satisfaction with having undergone surgery. Less than half of the participants found the decision of whether to undergo surgery difficult. However, the women who found it difficult had lower odds of being satisfied with their choice. Women who were generally satisfied with the support and information they received from the healthcare system had a 5.5 higher OR for being satisfied with having undergone surgery. Lack of pre-operative information has been associated with lower levels of satisfaction also in previous studies [16, 17]. Women who find the decision difficult may have an increased need for information and support, and a perceived lack of this may make the decision difficult. Both variables had a negative impact on satisfaction with having undergone surgery, and they may be related. Our estimates are based on retrospective data, and we cannot exclude that dissatisfaction with surgery leads to an interpretation of the process as difficult and the support they have received as insufficient. However, our combined observations indicate the importance of providing sufficient and clear information before surgery, while also ensuring that the women are fully involved in and confident about their decision prior to the procedure.

In the analysis, satisfaction levels for the outcome “satisfied with having undergone RRM” were dichotomized, and respondents who scored 6 or 7 were excluded to ensure a clear distinction between satisfied and not satisfied groups in the logistic regression analysis. However, this exclusion resulted in nearly as many participants being omitted as those classified as not satisfied. To investigate whether not excluding these respondents would lead to a better analysis, a multinomial regression analysis was also conducted including all three groups (scores 8–10, 6–7, and 1–5). The results indicated that the middle group (scores 6 and 7) lacked sufficient homogeneity to permit a coherent interpretation. These findings, in addition to the sensitivity analyses with respect to the cut points used in the standard logistic regression, showing consistent results with our primary analysis, support our decision to exclude the middle group in the primary analysis in order to maintain clear and interpretable results.

Previous studies have highlighted the relationship between expectations and satisfaction after surgery, and addressed the importance of assessing individual expectations and identify those who may have unrealistic expectations [18]. Although most participants were relieved after surgery and would have chosen the same again, 63 women (33%) expressed that the result was not as expected. However, only 13 would not have chosen the same operation again. There is likely a strong relationship between expectations and satisfaction in *BRCA1/2* carriers undergoing RRM, and they need precise and reliable information about expected results, complications and rehabilitation. However, the low number of women who would not have chosen the same again may reflect the women’s strong motivation for surgery. Moreover, 33% reported that the shape of the breasts was not satisfactory and 29% reported that the symmetry was not satisfactory. Even if they may have expected different cosmetic results, these aspects may not have led the women to regret their decision to undergo surgery. The respondents in our study have answered the questionnaire at different time points after surgery. Complications may arise several years after surgery, and satisfaction with surgical result may change over time. We therefore cannot exclude that our observations would be different if all had answered at the same time following surgery and at a shorter time after surgery. Prospective studies are needed to address these questions in more detail.

## Conclusion

In this study of RRM in *BRCA1/2* carriers without prior BC, we have observed that being a *BRCA1* carrier and having children are strongly associated with choosing to undergo RRM. The large majority of those who had chosen RRM were relieved, satisfied with their decision and would have chosen the same again. More than half of the participants thought the decision was easy, but those who found it difficult had lower odds of being satisfied. Feeling supported by healthcare providers in the decision-making process was strongly associated with greater satisfaction with having undergone surgery. Our observations underline that RRM is an important choice for many carriers. They also illustrate that information and support during the decision process is important for satisfaction following surgery. Studies are needed to further investigate whether satisfaction with having undergone surgery changes over time.

## Supplementary Information

Below is the link to the electronic supplementary material.

Supplementary file1 (DOCX 21 kb)

## Data Availability

Data will be made available upon reasonable request.

## Abbreviations

BC: Breast cancer
OC: Ovarian cancer
GT: Genetic testing
RRM: Risk-reducing mastectomy
