# Supplementary material for: Experiences with risk-reducing mastectomy in Norwegian BRCA1/2 carriers without prior breast cancer
Source: Fam Cancer. 2025 Jul 29;24(3):60. doi: 10.1007/s10689-025-00484-6 (PMC12307508; doi:10.1007/s10689-025-00484-6)
Supplement: Supplementary file 1 — Supplementary file1 (DOCX 21 kb) [file 10689_2025_484_MOESM1_ESM.docx]

**Experiences with Risk-Reducing Mastectomy in Norwegian *BRCA1/2* carriers without Prior Breast Cancer**

Hanne Kjensli Hyldebrandt¹^,^², Astrid Tenden Stormorken¹, Valeria Vitelli^3^, Amy Østertun Geirdal^4^ and Eli Marie Grindedal¹.

¹Department of Medical Genetics, Oslo University Hospital, Oslo, Norway. ²Institute of Clinical Medicine, University of Oslo, Oslo, Norway. ^3^Oslo Centre for Biostatistics and Epidemiology, Department of Biostatistics, Institute of Basic Medical Sciences, University of Oslo, Oslo, Norway. ^4^Department of Social Work, Child Welfare, and Social Policy, Faculty of Social Sciences, OsloMet – Oslo Metropolitan University, Oslo, Norway. Corresponding author: Hanne Kjensli Hyldebrandt, hanhyl@ous-hf.no.

**Supplementary tables:**

**Table S1: Reasons for choosing risk-reducing mastectomy (RRM)**

| Statements: |
| --- |
| - High likelihood of developing breast cancer  - Did not want to undergo breast cancer treatment  - Burdensome with breast exams  - Fear of dying from breast cancer  - Experienced relatives developing breast cancer  - Was recommended RRM by a geneticist/breast surgeon  - Other reasons |

**Table S2: Women who have chosen not to undergo risk-reducing mastectomy (RRM): Reasons for not choosing RRM**

| Statements: |
| --- |
| - Feel safe by attending screening  - Fear of surgery  - Want to breastfeed before potential surgery  - Medical reasons |

**Table S3: Questionnaire - The decision-making process.**

| **Experience of how the decision was made**.  The decision was difficult  What was difficult  **Received sufficient information/help/support from the genetic counselor**.  **Received sufficient information/help/support from the breast surgeon** | Independently/ In cooperation with health care professionals/ Health care professionals made the decision.  Yes/ No/ A little/ Do not know.  To decide *if* to do the surgery/ To decide *when* to do the surgery.  Yes/ No/ Do not know/ No need of information,help,support.  Yes/ No/ Do not know/ No need of information,help,support. |
| --- | --- |

**Table S4: Risk-reducing mastectomy (RRM) and satisfaction**

| Sufficient information before RRM  If no, would like more information regarding  Chosen the same operation  If no, would have chosen  Operative result as expected  Relief after RRM  Satisfactory size  Satisfactory shape  Satisfactory symmetry | Yes/ No/ Do not know/ Waiting for consultation before surgery  The procedure/ Rehabilitation after surgery/  The result/ Complications/ Other*  Yes/ No/ Do not know  Screening/ RRM, but without reconstruction/  Other type of implant/ Other  Yes/ No/ Do not know  Yes/ No/ Do not know  Yes/ No/ Do not know  Yes/ No/ Do not know  Yes/ No/ Do not know |
| --- | --- |

*Multiple answers possible

**Table S5: Sensitivity analyses**

Results of performed logistic regression analyses for the variable “ Satisfied with having undergone risk-reducing mastectomy” with different cutpoints.

1. **Cutpoints: Satisfied: 7-10, Not satisfied:1-6**

| Satisfied with having undergone RRM | Odds Ratio | p-value | 95% Confidence Interval |
| --- | --- | --- | --- |
| Satisfied with information/help from the healthcare during the decision-making process | 5.2 | 0.009 | (1.5-18.2) |
| Satisfied with information/ help from geneticists during the decision-making process | 0.5 | 0.355 | 0.1-2.4) |
| Satisfied with information/help from surgeon during the decision-making process | 2.1 | 0.310 | (0.5-8.6) |
| Difficult decision | 0.3 | 0.021 | (0.1-0.8) |

1. **Cutpoints: Satisfied: 6-10. Not satisfied:1-5**

| Satisfied with having undergone RRM | Odds Ratio | p-value | 95% Confidence Interval |
| --- | --- | --- | --- |
| Satisfied with information/help from the healthcare during the decision-making process | 6.7 | 0.004 | (1.8- 24.7) |
| Satisfied with information/ help from geneticists during the decision-making process | 0.5 | 0.378 | (0.1-2.5) |
| Satisfied with information/help from surgeon during the decision-making process | 2.3 | 0.262 | (0.5-9.9) |
| Difficult decision | 0.2 | 0.024 | (0.1-0.8) |

1. **Cutpoints: Satisfied: 8-10. Not satisfied: 1-5. Excluded from analysis: 6-7**

| Satisfied with having undergone RRM | Odds Ratio | p-value | 95% Confidence Interval |
| --- | --- | --- | --- |
| Satisfied with information/help from the healthcare during the decision-making process | 5.5 | 0.013 | (1.4-21.0) |
| Satisfied with information/ help from geneticists during the decision-making process | 0.6 | 0.598 | (0.1-3.5) |
| Satisfied with information/help from surgeon during the decision-making process | 2.8 | 0.180 | (0.6-12.2) |
| Difficult decision | 0.2 | 0.019 | (0.1-0.8) |
